# A Bibliometric Analysis on the Early Works of Dental Anxiety

**DOI:** 10.3390/dj11020036

**Published:** 2023-02-01

**Authors:** Andy Wai Kan Yeung

**Affiliations:** Oral and Maxillofacial Radiology, Applied Oral Sciences and Community Dental Care, Faculty of Dentistry, The University of Hong Kong, Hong Kong SAR, China; ndyeung@hku.hk

**Keywords:** anxiety, fear, dentistry, bibliometric, history, psychology

## Abstract

Dental anxiety has been a common phenomenon under investigation for decades. This report aimed to identify the historical roots of dental anxiety in the research literature. The literature database Web of Science Core Collection was searched to identify relevant papers on this theme. Cited reference analysis on the collected literature set was performed with CRExplorer, a dedicated bibliometric software. This analysis successfully identified the references dealing with dental anxiety in the late 1800s and early 1900s. They included essays that provided expert opinion on dental anxiety, reported semi-structured interviews to elucidate its underlying reasons, introduced psychometric scales to assess dental anxiety, and proposed theories and arguments from psychoanalytic aspects. Several references dealing with anxiety in general were also identified. To conclude, cited reference analysis was useful in revealing the historical origins of dental anxiety research. These cited references provided a concrete foundation to support subsequent dental anxiety research.

## 1. Introduction

Dental anxiety, or dental fear, makes patients more likely to avoid, postpone, and even cancel dental appointments [1]. A recent meta-analysis pooling data over 72,500 adults has computed a global prevalence of dental anxiety of 15.3%, with high and severe rates at 12.4% and 3.3%, respectively [2]. Being a relatively common phenomenon, dental anxiety became the focus of many research works, and it was identified as one of the recurring themes in the most cited papers in pediatric dentistry [3]. Recent papers from medicine, dentistry, and psychology [4,5,6] have coincidentally acknowledged the work by Coriat in 1946 as the first scientific description of dental anxiety [7]. Further to consulting academic scholars and historians who are experts of dental anxiety, could there be a systematic method to identify and confirm the origin(s) of the concept of dental anxiety?

The most intuitive method would be to search for the phrase “dental anxiety” and its variants in databases of the major literature and assume that Coriat (1946) [7] would appear as one of the most highly cited earlier works in the resultant list. However, it was found that it was not indexed by Web of Science as a primary document; no matter what the search string was, it would not be retrieved. To follow up, a cited reference search was conducted. It was much more restricted and allowed specific searches such as cited author, reference title, and publication year, but not specific phrases in the abstracts, keywords and so on, as cited references did not have complete bibliographic records. By searching Coriat as a cited author and publication year being 1946, it was found that the seminal work was indexed in two variants with a total of 20 citations, a very small number compared to highly cited dental anxiety publications, such as the paper reaffirming the reliability and validity of the well-known Corah’s Dental Anxiety Scale (DAS) [8] with over 400 citations. Meanwhile, Scopus and Dimensions indexed Coriat (1946) as a primary document, but with only 14 and 17 citations, respectively. Finally, PubMed also indexed Coriat (1946), but PubMed did not record citation counts.

Therefore, a cited reference analysis was performed with the CRExplorer software [9]. This analysis method had an advantage over the conventional citation analysis in this scenario, as the former could reveal references frequently cited by dental anxiety papers (that could be seldom cited by other papers and thus have a low total citation count). Through analyzing the cited references from a pre-defined literature set, one can plot a “reference publication year spectroscopy” (RPYS). An RPYS shows multiple waves along the timeline and illustrates in which years the cited references received much more citations relative to preceding and succeeding years (and thus suggesting that seminal works were published in those years) [10,11,12,13,14]. The primary aim of this study was to identify the historical root of dental anxiety by RPYS and confirm if it was Coriat (1946). Without prior knowledge, it would be logical to expect that the historical root of dental anxiety could be published any time before the invention of Corah’s DAS [15] (i.e., before 1969). Further to identifying the historical root, it was also very important to identify the seminal works published earlier in the field. These works might have proposed useful theories and provided innovative ideas to guide the investigations of future studies. Without a bibliometric analysis on the earlier works, they might not be easily come across and read by current researchers because of an overall low citation count and difficulty in accessing the main texts often stored online as scanned copies of the original print behind a paywall.

## 2. Materials and Methods

The Web of Science Core Collection electronic database was queried in late March 2021 to identify dental anxiety papers. The following search string was used: TOPIC: (“dental anxiety” OR “dental fear” OR “dental phobia” OR “odontophobia”). No additional filter was placed to restrict the search.

The full record and cited references of the resultant papers were exported to CRExplorer [9] to identify the most cited references. Default disambiguation procedures from the user manual were followed to unify the variants of cited references. This step was included because the cited reference data could often contain misspellings and errors. The positive and negative peaks shown in an RPYS indicate years when there was a deviation in the citation count relative to its 5-year median. Take the year 1969, when Corah’s DAS was invented, as an example. Within the downloaded literature set, references published in 1967–1971 were cited 72, 133, 770, 240, and 236 times, respectively. The 5-year median citation count was 236. Hence, references published in 1969, being cited 770 times collectively, were cited 534 times more than the 5-year median and created a positive peak with a magnitude of 534. Based on RPYS, positive peaks with a magnitude of >10 were noticed until 1969, and references were identified if they accounted for >10% of citations made to those peaks. These thresholds were set to filter for more representative references, similar to prior studies that excluded references with <10% contributions to a peak [16,17]. In order to search more thoroughly, recurring cited references (i.e., references being cited at least thrice) published before Coriat (1946) were identified and evaluated for their relevance to dental anxiety, without the need to fulfill the criteria set above. All identified cited references are included and listed in Table 1. None of them was excluded. Instead, the relevance of these cited references is evaluated and explained in Table 1. If they dealt with dentistry without considering the anxiety aspect of patients, or if they dealt with anxiety in general without a dental context, then they were considered irrelevant.

## 3. Results

The search yielded 2119 papers, and they had a total of 28,637 distinctive cited references after disambiguation. The oldest cited reference with 10 or more citations from these analyzed papers was by Pell and Gregory on their classification of impacted mandibular third molars [18] (12 citations). Similar to another recurring cited reference by Winter [19] (6 citations), they were not directly related to dental anxiety, but dental anxiety during third molar removal could be considered as a frequently investigated research topic.

Table 1 shows the references that were potentially historical roots of dental anxiety identified with the cited reference analysis methodology described above. Upon closer examination of their contents, six of them were highly relevant to dental anxiety. Three were essays on the authors’ expert opinion or summary on dental anxiety [7,20,21], two were introducing psychometric scales to measure dental anxiety (Corah’s DAS and Frankl’s behavior rating scale) [15,22], and one was a semi-structured interview to reveal underlying reasons for having dental anxiety [23]. Meanwhile, the author had no access to three references [24,25,26], and therefore their citing papers were accessed to identify their citation context. Steen (1891) [26] was cited by three papers to support the notions that child dental anxiety has been investigated for over a century and that it involves parental influence particularly from mothers [27,28,29]. Dinjian (1921) [24] was cited by three papers in the analyzed literature set. All three papers cited it in their introductory paragraph as a reference that attributed dental anxiety to the expectation of pain [30,31,32]. Richardson (1936) [25] was also cited by three papers, in two of which as a reference to reject the abovementioned connection between dental anxiety and pain expectation [30,33], and the other one as a reference to suggest a waste of professional time resulted from ineffective management of uncooperative patients [34].

**Table 1:** Details of potential historical roots of dental anxiety as revealed by the reference publication year spectrogram (RPYS) shown in Figure 2.

Year	Reference Number	Title	Number of Citations within the Dataset	Percentage of Citations Made to References of That Year	Relevance (V, Relevant; X, Irrelevant; ?, Unclear)
1891	[26]	Our relation to children	3	100.0%	(?) Potentially relevant to child dental anxiety (no access to the publication).
1920	[35]	Conditioned emotional reactions	3	75.0%	(X) Reported experimental findings of conditioned fear responses in a male infant. Conditions were not related to dental anxiety (e.g., confronted him suddenly with a white rat, rabbit, dog, etc.).
1921	[24]	The psychic factor in dental practice	3	100.0%	(?) Potentially relevant to dental anxiety (no access to the publication).
1923	[20]	Psychology in dentistry	4	57.1%	(V) Briefly commented that dental fear could be unexplainable, anticipatory of pain and serious problems, and related to confidence towards the dentist.
1926	[19]	The principles of exodontia as applied to the impacted third molar (Book)	6	75.0%	(X) Introduced a classification for impacted third molars.
1929	[36]	Progressive relaxation (1st ed; Book)	5	50.0%	(X) Discussed progressive muscle relaxation, a strategy that may be adopted to manage anxiety.
1933	[18]	Impacted mandibular third molars: classification and modified techniques for removal	12	100.0%	(X) Introduced a classification for impacted third molars.
1936	[25]	Fear—a dental problem	3	50.0%	(?) Potentially relevant to dental anxiety (no access to the publication).
1938	[37]	Progressive relaxation (2nd ed; Book)	8	44.4%	(X) Discussed progressive muscle relaxation, a strategy that may be adopted to manage anxiety.
	[38]	Studies on dental caries: I. Dental status and dental needs of elementary school children	4	22.2%	(X) Investigated dental caries and dental needs of elementary school children.
	[21]	The psychology of fear in dentistry	3	16.7%	(V) Explained in detail that dental fear could be attributable to eight aspects, from prior unpleasant dental experience to symbolic associations.
1939	[39]	A stimulus-response analysis of anxiety and its role as a reinforcing agent	5	50.0%	(X) Reviewed the literature and argued that anxiety should be a learned (conditioned) response anticipatory of injury or pain. Moreover, anxiety could be irrationally disproportionate to the extent of danger.
1946	[7]	Dental anxiety; fear of going to the dentist	11	73.3%	(V) Articulated dental anxiety from a psychoanalysis view. It could be anticipatory of pain and danger. It could also be neurotic, meaning unconsciously perceiving the treatment or removal of a tooth as symbolic castration.
1951	[40]	Coefficient alpha and the internal structure of tests	16	64.0%	(X) Introduced a test score reliability coefficient known as Cronbach’s alpha.
1954	[23]	An empirical study of the etiology of dental fears	51	75.0%	(V) Performed semi-structured interviews on 30 adults. Half of them displayed intense emotional patterns in the dental office and formed the fearful group, and the other half formed the control group. Results showed that the fearful group had a significantly higher ratio of unfavorable family dental experience and attitude toward dentistry. No significant differences were found for pain tolerance, traumatic healthcare experience, general anxiety level, trouble with authority, appearance, and psychoanalytic factors (orality and dependency).
1958	[41]	The public looks at dental care	32	44.4%	(X) Reported a public health survey. Fear of dentists and pain were among the commonest reasons for both not seeing a dentist more often and not having needed dental care.
	[42]	Psychotherapy by reciprocal inhibition (Book)	16	22.2%	(X) Introduced reciprocal inhibition. By teaching patients to relax and confront the fear via imagery manipulations in behavioral treatment, the new behavior could replace the old one.
1962	[22]	Should the parent remain with the child in the dental operatory?	71	53.8%	(V) Introduced Frankl’s behavior rating scale to assess child dental anxiety.
1965	[43]	The development of a scale to measure fear	48	49.5%	(X) Introduced a fear scale, Fear Survey Schedule-II, to measure fear in general.
1966	NA				
1969	[15]	Development of a Dental Anxiety Scale	547	71.0%	(V) Introduced the well-known Corah’s Dental Anxiety Scale.

The listed cited references either (a) accounted for >10% of citations in positive peaks prior to 1969 with magnitude >50 in the RPYS, or (b) received at least three citations and published before 1946. NA means there was no reference meeting the pre-defined criteria in that year.

**Figure 1:**
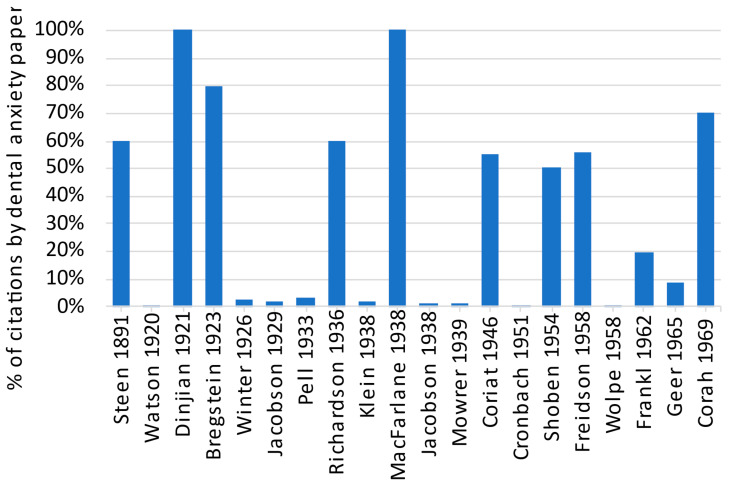
Ratio of citations received by the selected cited references from dental anxiety papers. Steen 1891 [26]; Watson 1920 [35]; Dinjian 1921 [24]; Bregstein 1923 [20]; Winter 1926 [19]; Jacobson 1929 [36]; Pell 1933 [18]; Richardson 1936 [25]; Klein 1938 [37]; MacFarlane 1938 [38]; Jacobson 1938 [21]; Mowrer 1939 [39]; Coriat 1946 [7]; Cronbach 1951 [40]; Shoben 1954 [23]; Freidson 1958 [41]; Wolpe 1958 [42]; Frankl 1962 [22]; Geer 1965 [43]; Corah 1969 [15].

Further to manually determining if the cited references selected above were relevant to dental anxiety or not, the ratios of citations received by the cited references from dental anxiety papers (i.e., the collection of 2119 papers retrieved from the search) were computed (Figure 1). When an arbitrary and intuitive threshold of 50% was set, one could observe that the cited references deemed relevant and potentially relevant were all identified except [22] (20% only). On the other hand, one cited reference passing the threshold [41] (58%) reported a public health survey mentioning that fear of dentists and pain were among the commonest reasons for not seeing a dentist and having the required dental care. This work was deemed not directly relevant to dental anxiety by the author, as the survey covered a wide range of areas concerning dental care.

**Figure 2:**
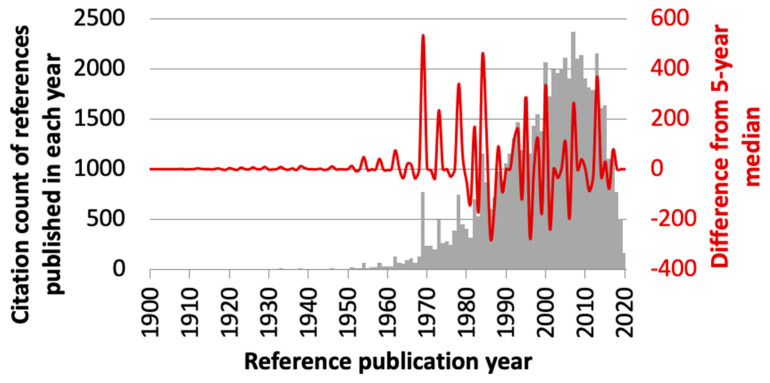
Reference publication year spectrogram (RPYS) created by analyzing 2119 papers on dental anxiety. The grey bar chart shows the citation count of references published in each publication year. The red wave form shows the citation differences from the 5-year median. Take the year 1969 as one example. The numbers of citations gained by references published in 1967–1971 were 72, 133, 770, 240, and 236, respectively. The 5-year median was 236. The peak in 1969, therefore, had a magnitude of 770 − 236 = 534. By 1969, there were 8 peaks with magnitude >50, namely, 1938, 1946, 1951, 1954, 1958, 1962, 1965–1966, and 1969.

Figure 2 shows an RPYS spectrogram. Before the year 1969, there were seven peaks (1938, 1946, 1951, 1954, 1958, 1962, 1965–1966). At the peak that corresponds to the year 1938, MacFarlane (1938) [21] was identified. It explained dental anxiety from eight aspects, namely, (1) the memory of previous unpleasant dental experience, (2) loss of power, (3) fear of the unknown, (4) memory of unpleasant experience (not dental), (5) general mental tendencies, inherited or acquired, (6) specific stimuli, (7) emotional sympathy, and (8) symbolic association. This essay was considerably longer than an earlier account by Bregstein (1923) [20] (nine vs. three pages).

## 4. Discussion

This cited reference analysis successfully identified the papers that introduced the very first psychometric scales to assess dental anxiety. It also found that the historical roots of dental anxiety could be traced back to the late 1800s and early 1900s, such as [26], [24], and [20]. It was confirmed that Coriat (1946) was not the earliest historical root of dental anxiety, but it was the earliest cited reference that concerned dental anxiety and was published in a psychology journal. Since some very old publications do not have an abstract section or their abstracts are not indexed by the literature databases, a simple search by keywords may be unable to identify all of them.

The contemporary understanding of dental anxiety is that it is a multi-faceted psychological issue. It can be divided into trait and state components. The former deals with personality traits and is more stable over time, and the latter is situational, anticipatory, and related to treatment [44]. Dental anxiety is of high interest to the general public, and there are multiple online videos including patient testimonials that explain how to manage it [45]. In academia, numerous pharmacological and non-pharmacological management methods have been tested, with mixed results [46]. Especially during the COVID-19 pandemic, dental clinics were deemed by patients to be a risky environment with danger of being infected due to mask removal and aerosol generation [47]. It was found that patients had lower dental anxiety level after being vaccinated [47]. For a comprehensive literature review on the etiology and maintenance of dental anxiety in both adults and children, readers may refer to [48]. Dental anxiety also presents with four aspects of symptoms, namely, the emotional, physical, cognitive, and behavioral [1,49]. As such, multiple dental anxiety scales have been developed with different conceptual and theoretical bases [1]. The contemporary conceptual and theoretical basis was largely built upon some of the earlier works such as [20] published in 1923. For example, the foci on pain-free experience and trust building were consistent with the notion from [20] that dental fear could be due to anticipation of pain and serious problems, and related to confidence towards the dentist.

One facet (or proposition) of dental anxiety that is not widely picked up and developed in the dental field is symbolic association, as extensively elaborated by [7]. In the field of psychoanalysis, a tooth can be perceived as a phallic symbol. The treatment or loss of a tooth may be a castration symbol, and therefore going to the dentist may trigger the fear of castration [7]. This thought originated from Sigmund Freud’s *The Interpretation of Dreams* [50]. Coriat substantiated this claim by referring to the extraction of the maxillary incisors as “a puberty rite in place of circumcision” among prehistoric man and primitive tribes [7,51]. This proposition was dismissed by [21], occasionally mentioned by the psychology field [52,53], and did not gain momentum in the dental profession. This phenomenon was consistent with the finding that the influence of Freud was generally in decline [54]. Regardless, the notion of connecting tooth extraction to castration anxiety should probably be abandoned in contemporary dentistry and psychology.

This study had several limitations. First, it was not possible to gain access to the very old publications. This implied that some theories and arguments might have been developed or proposed much earlier than modern scholars thought, and hence some earlier scholars might not be acknowledged properly. Second, the literature databases, including Web of Science, may entirely miss the reference lists of the very old publications. Moreover, findings and concepts with a long history may become common knowledge, and thus the source articles are no longer cited, a phenomenon called obliteration by incorporation [54,55,56]. These issues might underestimate the scientific impact of the cited references.

To conclude, this scientometric analysis on dental anxiety demonstrated that cited reference analysis was a pragmatic method to identify relevant and important papers published in the distant past. The academic interest of dental anxiety could be dated back to the late 1800s and early 1900s, with a notable historical root being Steen (1891) published in a dental journal. Meanwhile, Coriat (1946) was identified as one of first historical roots from the psychology field. Cited reference analysis can be useful for multiple purposes, such as to identify its historical development or conduct a systematic review. This work may better inform dentists and researchers that dental anxiety is a subject tangling between dentistry and psychology, with a long history. Finally, dental anxiety is both an important issue for patients and dentistry in general in the modern era, requiring continuing efforts to reduce and manage this psychological state.

## Data Availability

All data are available in the manuscript.
